# *r*-1,*t*-3-Bis[4-(di­methyl­amino)­phen­yl]-*c*-2,*t*-4-bis­(pyridin-4-yl)cyclo­butane

**DOI:** 10.1107/S1600536814002311

**Published:** 2014-02-15

**Authors:** Shuguang Zhang, Junpeng Zhuang

**Affiliations:** a Department of Organic Chemistry, Faculty of Science, Beijing University of Chemical Technology, Beijing 100029, People’s Republic of China

## Abstract

The title compound, C_30_H_32_N_4_, was synthesized by the photodimerization of *trans*-4-{2-[4-(di­methyl­amino)­phen­yl]ethen­yl}pyridine in benzene upon irradiation with UV light. This photodimer has a puckered cyclo­butane ring with the four aryl substituents in an *r*-1,*t*-2,*c*-3,*t* conformation. The puckering angle of the cyclo­butane ring is 32.22 (7)°, which is the largest among reported tetra­aryl-substituted cyclo­butanes. In the crystal, the mol­ecules form a hollow, one-dimensional structure extending parallel to the *c* axis *via* two different pairs of C—H⋯π inter­actions.

## Related literature   

For the photodimerization of styrylpryidines, see: Horner & Hünig (1982 ▶); Quina & Whitten (1975 ▶); Zhang, Zhang, Zheng, Shen & Zhuang (2000 ▶). For the single-crystal structures of tetra­aryl cyclo­butanes and related mol­ecules, see: Busetti *et al.* (1980 ▶); Coe *et al.* (2005 ▶); Li *et al.* (2007 ▶); Zhang *et al.* (1998 ▶); Zhang, Zhang, Zheng, Wang & Zhao (2000 ▶); Zhuang & Zheng (2002 ▶). For the synthesis of the monomer, see: Wang *et al.* (2005 ▶).
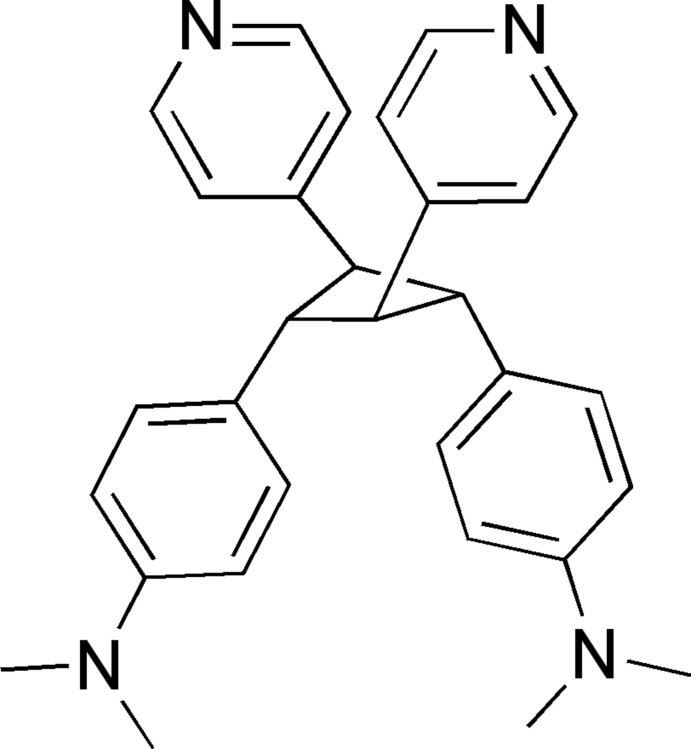


## Experimental   

### 

#### Crystal data  

C_30_H_32_N_4_
*M* *_r_* = 448.60
Monoclinic, 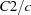

*a* = 23.166 (5) Å
*b* = 11.003 (2) Å
*c* = 9.6330 (19) Å
β = 91.67 (3)°
*V* = 2454.4 (8) Å^3^
*Z* = 4
Mo *K*α radiation
μ = 0.07 mm^−1^
*T* = 113 K
0.24 × 0.20 × 0.16 mm

#### Data collection  

Rigaku Saturn 70 CCD diffractometer
Absorption correction: multi-scan (*CrystalClear*; Rigaku, 2009 ▶) *T*_min_ = 0.983, *T*_max_ = 0.989
14966 measured reflections
2934 independent reflections
2335 reflections with *I* > 2σ(*I*)
*R*_int_ = 0.043

#### Refinement  

*R*[*F* ^2^ > 2σ(*F* ^2^)] = 0.045
*wR*(*F* ^2^) = 0.121
*S* = 1.09
2934 reflections
157 parameters
H-atom parameters constrained
Δρ_max_ = 0.24 e Å^−3^
Δρ_min_ = −0.26 e Å^−3^

### 

Data collection: *CrystalClear* (Rigaku, 2009 ▶); cell refinement: *CrystalClear*; data reduction: *CrystalClear*; program(s) used to solve structure: *SHELXS97* (Sheldrick, 2008 ▶); program(s) used to refine structure: *SHELXL97* (Sheldrick, 2008 ▶); molecular graphics: *SHELXTL* (Sheldrick, 2008 ▶); software used to prepare material for publication: *SHELXTL*.

## Supplementary Material

Crystal structure: contains datablock(s) global, I. DOI: 10.1107/S1600536814002311/mw2110sup1.cif

Structure factors: contains datablock(s) I. DOI: 10.1107/S1600536814002311/mw2110Isup2.hkl

Supporting information file. DOI: 10.1107/S1600536814002311/mw2110Isup3.cml

CCDC reference: 984465

Additional supporting information:  crystallographic information; 3D view; checkCIF report

## Figures and Tables

**Table 1:** Hydrogen-bond geometry (Å, °) *Cg*1 and *Cg*2 are the centroids of the C8–C13 and N1/C1–C5 rings, respectively.

*D*—H⋯*A*	*D*—H	H⋯*A*	*D*⋯*A*	*D*—H⋯*A*
C15—H15*B*⋯*Cg*1^i^	0.96	2.82	3.679 (2)	149
C12—H12⋯*Cg*2^i^	0.96	3.12	4.104 (2)	161

Symmetry code: (i) 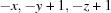.

**Fig. 1.**
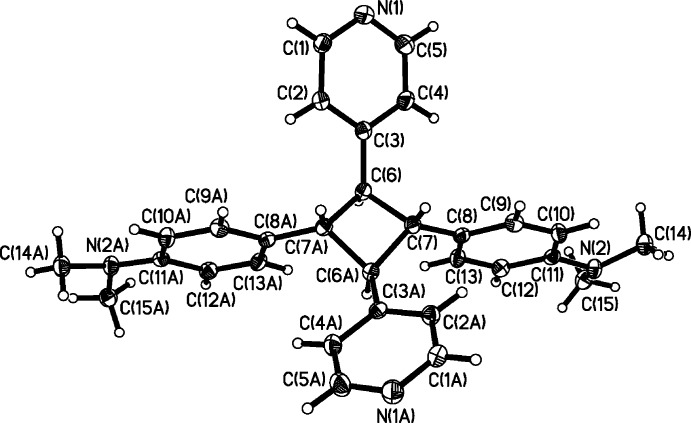
Molecular structure of 2A. Displacement ellipsoids are drawn at the 50% probability level.

**Fig. 2.**
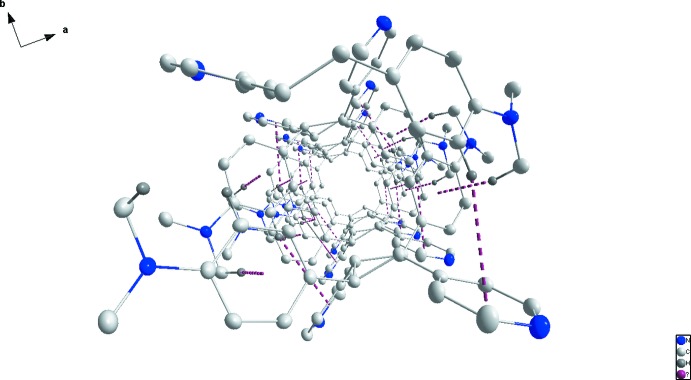
Packing diagram of 2A viewed along c axis.

**Fig. 3.**
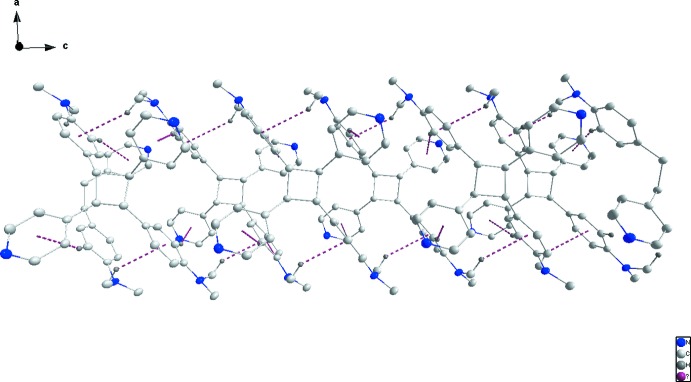
Packing diagram of 2A viewed along b axis.

## supplementary crystallographic information

### 1. Comment

The photodimerization of styrylpyridines has been studied extensively in the past few decades (Horner & Hünig, 1982; Quina & Whitten, 1975; Zhang, Zhang, Zheng, Shen & Zhuang, 2000). As the protonation of the pyridine ring increases the solubility and dipolar interactions of styrylpyridines in water, most photodimerization reactions were carried out in acidic aqueous solution. The main tetraaryl substituted cyclobutane photodimers usually have head-to-tail central symmetric structures. However, the photodimerization of *trans*-4-[2-(4-dimethylaminophenyl)ethenyl]pyridine (A) in acidic aqueous solution failed (Zhang, Zhang, Zheng, Shen & Zhuang, 2000). Herein, the photodimerization of A was carried out in benzene and the photodimer (2A, Fig. 1) was successfully synthesized in 37% yield.

The structure of 2A shows that the four aryl substituents adopt the *r*-1,*t*-2,*c*-3,*t*-4 conformation, whereas the styrylpyridine photodimers synthesized in acidic aqueous solution adopt the head-to-tail *r*-1,*c*-2,*t*-3,*t*-4 conformation. In addition, the photodimer of 2-[2-(4-dimethylaminophenyl)ethenyl]benzoxazole synthesized in acetonitrile also adopts the head-to-tail *r*-1,*c*-2,*t*-3,*t*-4 conformation (Li *et al.*, 2007). This difference is ascribed to solvent effects. The steric hindrance of the dimethylamino groups prevents A to align in a parallel manner in the non-polar benzene solvent, resulting in the all *trans* conformation of the adjacent aryl groups in 2A.

Several styrylpyridine photodimers such as *r*-1,*c*-2,*t*-3,*t*-4–1,3-bis[2-(4-*R*-phenyl)]-2,4-di(pyridin-4-yl)cyclobutane (*R*=Cl, CH_3_ and C_6_H_5_) have been reported (Busetti *et al.*, 1980; Zhang *et al.*, 1998; Zhang, Zhang, Zheng, Wang & Zhao, 2000). The average dihedral angles of the cyclobutane rings are 19.2, 24.6 and 16.4°, respectively. In addition, the *trans*-head-to-head photodimer of 1-(4-methoxyphenyl)-2-(5-phenyl-1,3,4-oxadiazolyl)ethene also has a puckered cyclobutane ring with a dihedral angle of 30° (Zhuang & Zheng, 2002). Though the adjacent aryl groups of 2A adopt the all *trans* conformation, the dihedral angle of the cyclobutane ring is 32.22 (7)° which is the largest one among reported tetraaryl substituted cyclobutanes.

The C6—C7 and C6—C7A bond distances are 1.5594 (16) Å and 1.5519 (16) Å, and the C3—C6 and C7—C8 bond distances are 1.4962 (18) Å and 1.5011 (16) Å, which are similar to the corresponding bond distances in other tetraaryl substituted cyclobutanes (Zhang *et al.*, 1998; Zhang, Zhang, Zheng, Wang & Zhao, 2000). The two phenyl rings (C8—C9—C10—C11—C12—C13 and C8A—C9A—C10A—C11A—C12A—C13A) are almost coplanar with the dihedral angle between them being only 1.78 (7)°.

The dimethylamino plane and the phenyl ring (C8—C9—C10—C11—C12—C13) are not coplanar, the torsion angle (C14—N2—C11—C10) is 17.48 (18)° which is larger than that in *c*-2, *t*-4-bis(2-benzoxazol-2-yl)-*r*-1,*t*-3-bis[4-(dimethylamino)phenyl]cyclobutane (Li *et al.*, 2007). The distances of the two methyl groups (C14 and C15) from the mean plane of the phenyl ring are 0.2629 (22) and 0.2335 (22) Å respectively, indicating that the lone electron pair of the N atom is not completely conjugated with the phenyl ring. While the dimethylaminophenyl ring in *trans*-4-[(4-dimethylaminophenyl)ethenyl]-*N*-methylquinolinium *p*-toluenesulfonate monohydrate (Coe *et al.*, 2005) is essentially planar because of the strong *p*-π conjugation between the dimethylamino group and the planar diarylethene molecule.

As shown in Figure 2, the molecules of 2A pack with each other to form a hollow, one-dimensional structure along the *c* axis. This arrangement appears to be directed by two sets of C—H···π interactions with that involving H15B stronger than that using H12 (Table 1).

### 2. Experimental

A was synthesized according to the literature (Wang *et al.*, 2005) and 1.97 g (8.78 mmol) was dissolved in 200 mL of benzene and irradiated with a water-cooled 125 W medium-pressure mercury lamp which was immersed in the solution. After irradiation for about 30 h, the solvent was evaporated to dryness and the crude product was separated by column chromatography (ethyl acetate: dichloromethane = 1: 1) to give 0.73 g of colorless crystals of 2 A. Yield, 37%; ^1^H NMR (CDCl_3_): *δ* 8.35 (d, 4H, *J*=4.8 Hz), 7.00 (d, 4H, *J*=4.8 Hz), 6.94 (d, 4H, *J*=6.4 Hz), 6.53 (d, 4H, *J*=6.4 Hz), 4.38 (d, 2H, *J*=9.0 Hz), 74.28 (d, 2H, *J*=9.0 Hz) p.p.m.. The single-crystal of 2A, suitable for X-ray analysis, was grown by slow evaporation of a methanol solution of 2A.

### 3. Refinement

H atoms were placed in calculated positions [C—H = 0.93–0.97 Å] and allowed to ride on the parent atoms, with *U*_iso_ values constrained to be 1.2*U*_eq_ of the parent atom.

### Crystal data

**Table:**

C_30_H_32_N_4_	*F*(000) = 960
*M**_r_* = 448.60	*D*_x_ = 1.214 Mg m^−^^3^
Monoclinic, *C*2/*c*	Mo *K*α radiation, λ = 0.71073 Å
Hall symbol: -C 2yc	Cell parameters from 2660 reflections
*a* = 23.166 (5) Å	θ = 1.8–27.9°
*b* = 11.003 (2) Å	µ = 0.07 mm^−^^1^
*c* = 9.6330 (19) Å	*T* = 113 K
β = 91.67 (3)°	Block, colourless
*V* = 2454.4 (8) Å^3^	0.24 × 0.20 × 0.16 mm
*Z* = 4	

### Data collection

**Table:**

Rigaku Saturn 70 CCD diffractometer	2934 independent reflections
Radiation source: rotating anode	2335 reflections with *I* > 2σ(*I*)
Confocal monochromator	*R*_int_ = 0.043
ω scans	θ_max_ = 27.9°, θ_min_ = 1.8°
Absorption correction: multi-scan (*CrystalClear*; Rigaku, 2009)	*h* = −30→30
*T*_min_ = 0.983, *T*_max_ = 0.989	*k* = −14→14
14966 measured reflections	*l* = −12→12

### Refinement

**Table:**

Refinement on *F*^2^	Secondary atom site location: difference Fourier map
Least-squares matrix: full	Hydrogen site location: inferred from neighbouring sites
*R*[*F*^2^ > 2σ(*F*^2^)] = 0.045	H-atom parameters constrained
*wR*(*F*^2^) = 0.121	*w* = 1/[σ^2^(*F*_o_^2^) + (0.0689*P*)^2^] where *P* = (*F*_o_^2^ + 2*F*_c_^2^)/3
*S* = 1.09	(Δ/σ)_max_ < 0.001
2934 reflections	Δρ_max_ = 0.24 e Å^−^^3^
157 parameters	Δρ_min_ = −0.26 e Å^−^^3^
0 restraints	Extinction correction: *SHELXL97* (Sheldrick, 2008), Fc^*^=kFc[1+0.001xFc^2^λ^3^/sin(2θ)]^-1/4^
Primary atom site location: structure-invariant direct methods	Extinction coefficient: 0.0115 (17)

### Special details

**Table:**

Geometry. All esds (except the esd in the dihedral angle between two l.s. planes) are estimated using the full covariance matrix. The cell esds are taken into account individually in the estimation of esds in distances, angles and torsion angles; correlations between esds in cell parameters are only used when they are defined by crystal symmetry. An approximate (isotropic) treatment of cell esds is used for estimating esds involving l.s. planes.
Refinement. Refinement of *F*^2^ against ALL reflections. The weighted *R*-factor *wR* and goodness of fit *S* are based on *F*^2^, conventional *R*-factors *R* are based on *F*, with *F* set to zero for negative *F*^2^. The threshold expression of *F*^2^ > σ(*F*^2^) is used only for calculating *R*-factors(gt) *etc*. and is not relevant to the choice of reflections for refinement. *R*-factors based on *F*^2^ are statistically about twice as large as those based on *F*, and *R*- factors based on ALL data will be even larger.

### Fractional atomic coordinates and isotropic or equivalent isotropic displacement parameters (Å^2^)

**Table:**

	*x*	*y*	*z*	*U*_iso_*/*U*_eq_	
N1	−0.12781 (4)	0.08386 (9)	0.34546 (11)	0.0261 (3)	
N2	0.21597 (4)	0.48468 (8)	0.38134 (10)	0.0201 (3)	
C1	−0.14278 (5)	0.08343 (11)	0.47858 (13)	0.0215 (3)	
H1	−0.1758	0.0408	0.5017	0.026*	
C2	−0.11207 (5)	0.14263 (10)	0.58397 (12)	0.0191 (3)	
H2	−0.1241	0.1375	0.6750	0.023*	
C3	−0.06316 (5)	0.20975 (10)	0.55322 (12)	0.0166 (3)	
C4	−0.04739 (5)	0.21074 (11)	0.41486 (12)	0.0233 (3)	
H4	−0.0151	0.2541	0.3884	0.028*	
C5	−0.08012 (6)	0.14681 (12)	0.31670 (13)	0.0289 (3)	
H5	−0.0683	0.1477	0.2253	0.035*	
C6	−0.02904 (5)	0.27871 (10)	0.66140 (12)	0.0170 (3)	
H6	−0.0342	0.3661	0.6456	0.020*	
C7	0.03660 (4)	0.25051 (10)	0.68241 (11)	0.0165 (3)	
H7	0.0421	0.1627	0.6722	0.020*	
C8	0.08169 (5)	0.31420 (10)	0.60052 (11)	0.0165 (3)	
C9	0.12213 (5)	0.24764 (10)	0.52824 (12)	0.0191 (3)	
H9	0.1200	0.1633	0.5298	0.023*	
C10	0.16550 (5)	0.30229 (10)	0.45398 (12)	0.0204 (3)	
H10	0.1914	0.2541	0.4065	0.024*	
C11	0.17092 (5)	0.42906 (10)	0.44948 (11)	0.0172 (3)	
C12	0.12953 (5)	0.49722 (10)	0.52068 (12)	0.0196 (3)	
H12	0.1312	0.5816	0.5186	0.023*	
C13	0.08643 (5)	0.44051 (11)	0.59377 (12)	0.0193 (3)	
H13	0.0599	0.4881	0.6397	0.023*	
C14	0.24760 (5)	0.41298 (11)	0.28156 (13)	0.0240 (3)	
H14A	0.2213	0.3836	0.2103	0.036*	
H14B	0.2658	0.3453	0.3281	0.036*	
H14C	0.2766	0.4628	0.2405	0.036*	
C15	0.21211 (5)	0.61429 (10)	0.35107 (13)	0.0230 (3)	
H15A	0.2091	0.6588	0.4363	0.034*	
H15B	0.1786	0.6297	0.2927	0.034*	
H15C	0.2461	0.6398	0.3043	0.034*	

### Atomic displacement parameters (Å^2^)

**Table:**

	*U*^11^	*U*^22^	*U*^33^	*U*^12^	*U*^13^	*U*^23^
N1	0.0243 (6)	0.0315 (6)	0.0226 (6)	−0.0049 (5)	0.0012 (4)	−0.0045 (5)
N2	0.0177 (5)	0.0189 (5)	0.0240 (5)	−0.0004 (4)	0.0060 (4)	0.0021 (4)
C1	0.0181 (6)	0.0226 (6)	0.0239 (6)	−0.0010 (5)	0.0020 (5)	−0.0015 (5)
C2	0.0185 (6)	0.0205 (6)	0.0185 (6)	0.0006 (4)	0.0026 (5)	−0.0006 (5)
C3	0.0156 (5)	0.0155 (5)	0.0186 (6)	0.0037 (4)	0.0004 (4)	0.0003 (4)
C4	0.0193 (6)	0.0292 (7)	0.0215 (6)	−0.0045 (5)	0.0033 (5)	0.0011 (5)
C5	0.0278 (7)	0.0404 (8)	0.0185 (6)	−0.0058 (6)	0.0036 (5)	−0.0033 (6)
C6	0.0144 (5)	0.0190 (6)	0.0176 (6)	−0.0004 (4)	0.0009 (4)	0.0006 (5)
C7	0.0155 (6)	0.0158 (6)	0.0181 (6)	0.0014 (4)	0.0015 (5)	−0.0004 (4)
C8	0.0137 (5)	0.0204 (6)	0.0154 (6)	−0.0003 (4)	−0.0006 (4)	0.0003 (5)
C9	0.0201 (6)	0.0158 (6)	0.0215 (6)	−0.0002 (4)	0.0023 (5)	0.0004 (5)
C10	0.0193 (6)	0.0198 (6)	0.0224 (6)	0.0019 (5)	0.0057 (5)	−0.0020 (5)
C11	0.0146 (5)	0.0199 (6)	0.0173 (6)	−0.0006 (4)	0.0000 (4)	0.0019 (5)
C12	0.0193 (6)	0.0159 (6)	0.0234 (6)	0.0009 (4)	0.0005 (5)	0.0012 (5)
C13	0.0157 (5)	0.0187 (6)	0.0237 (6)	0.0027 (4)	0.0031 (5)	−0.0002 (5)
C14	0.0210 (6)	0.0267 (7)	0.0247 (7)	−0.0009 (5)	0.0076 (5)	0.0015 (5)
C15	0.0213 (6)	0.0214 (6)	0.0264 (7)	−0.0023 (5)	0.0034 (5)	0.0047 (5)

### Geometric parameters (Å, º)

**Table:**

N1—C1	1.3381 (15)	C7—C6^i^	1.5512 (15)
N1—C5	1.3396 (16)	C7—H7	0.9800
N2—C11	1.3908 (14)	C8—C9	1.3918 (15)
N2—C14	1.4576 (15)	C8—C13	1.3958 (16)
N2—C15	1.4579 (14)	C9—C10	1.3874 (15)
C1—C2	1.3853 (17)	C9—H9	0.9300
C1—H1	0.9300	C10—C11	1.4013 (16)
C2—C3	1.3916 (15)	C10—H10	0.9300
C2—H2	0.9300	C11—C12	1.4109 (16)
C3—C4	1.3923 (16)	C12—C13	1.3867 (16)
C3—C6	1.4961 (16)	C12—H12	0.9300
C4—C5	1.3863 (17)	C13—H13	0.9300
C4—H4	0.9300	C14—H14A	0.9600
C5—H5	0.9300	C14—H14B	0.9600
C6—C7^i^	1.5512 (15)	C14—H14C	0.9600
C6—C7	1.5593 (15)	C15—H15A	0.9600
C6—H6	0.9800	C15—H15B	0.9600
C7—C8	1.5008 (15)	C15—H15C	0.9600
			
C1—N1—C5	116.00 (11)	C9—C8—C13	116.46 (10)
C11—N2—C14	118.14 (10)	C9—C8—C7	120.41 (10)
C11—N2—C15	118.84 (9)	C13—C8—C7	123.12 (10)
C14—N2—C15	115.26 (9)	C10—C9—C8	122.55 (11)
N1—C1—C2	123.96 (11)	C10—C9—H9	118.7
N1—C1—H1	118.0	C8—C9—H9	118.7
C2—C1—H1	118.0	C9—C10—C11	120.95 (10)
C1—C2—C3	119.84 (11)	C9—C10—H10	119.5
C1—C2—H2	120.1	C11—C10—H10	119.5
C3—C2—H2	120.1	N2—C11—C10	121.45 (10)
C2—C3—C4	116.48 (11)	N2—C11—C12	121.70 (10)
C2—C3—C6	122.53 (10)	C10—C11—C12	116.83 (10)
C4—C3—C6	120.99 (10)	C13—C12—C11	121.14 (11)
C5—C4—C3	119.70 (11)	C13—C12—H12	119.4
C5—C4—H4	120.2	C11—C12—H12	119.4
C3—C4—H4	120.2	C12—C13—C8	122.04 (10)
N1—C5—C4	124.00 (11)	C12—C13—H13	119.0
N1—C5—H5	118.0	C8—C13—H13	119.0
C4—C5—H5	118.0	N2—C14—H14A	109.5
C3—C6—C7^i^	120.12 (9)	N2—C14—H14B	109.5
C3—C6—C7	118.86 (9)	H14A—C14—H14B	109.5
C7^i^—C6—C7	88.36 (9)	N2—C14—H14C	109.5
C3—C6—H6	109.3	H14A—C14—H14C	109.5
C7^i^—C6—H6	109.3	H14B—C14—H14C	109.5
C7—C6—H6	109.3	N2—C15—H15A	109.5
C8—C7—C6^i^	121.09 (10)	N2—C15—H15B	109.5
C8—C7—C6	121.99 (9)	H15A—C15—H15B	109.5
C6^i^—C7—C6	87.07 (9)	N2—C15—H15C	109.5
C8—C7—H7	108.2	H15A—C15—H15C	109.5
C6^i^—C7—H7	108.2	H15B—C15—H15C	109.5
C6—C7—H7	108.2		
			
C5—N1—C1—C2	−0.30 (18)	C6—C7—C8—C9	−126.96 (12)
N1—C1—C2—C3	1.49 (18)	C6^i^—C7—C8—C13	−53.68 (15)
C1—C2—C3—C4	−1.27 (16)	C6—C7—C8—C13	54.16 (16)
C1—C2—C3—C6	178.20 (11)	C13—C8—C9—C10	0.62 (18)
C2—C3—C4—C5	0.03 (17)	C7—C8—C9—C10	−178.34 (10)
C6—C3—C4—C5	−179.44 (11)	C8—C9—C10—C11	0.61 (19)
C1—N1—C5—C4	−1.05 (19)	C14—N2—C11—C10	17.54 (17)
C3—C4—C5—N1	1.2 (2)	C15—N2—C11—C10	165.43 (11)
C2—C3—C6—C7^i^	16.79 (16)	C14—N2—C11—C12	−164.33 (11)
C4—C3—C6—C7^i^	−163.77 (10)	C15—N2—C11—C12	−16.44 (17)
C2—C3—C6—C7	123.18 (12)	C9—C10—C11—N2	176.66 (11)
C4—C3—C6—C7	−57.38 (15)	C9—C10—C11—C12	−1.55 (17)
C3—C6—C7—C8	88.20 (13)	N2—C11—C12—C13	−176.89 (11)
C7^i^—C6—C7—C8	−147.91 (9)	C10—C11—C12—C13	1.32 (17)
C3—C6—C7—C6^i^	−146.52 (8)	C11—C12—C13—C8	−0.12 (18)
C7^i^—C6—C7—C6^i^	−22.63 (11)	C9—C8—C13—C12	−0.86 (17)
C6^i^—C7—C8—C9	125.21 (12)	C7—C8—C13—C12	178.07 (11)

Symmetry code: (i) −*x*, *y*, −*z*+3/2.

### Hydrogen-bond geometry (Å, º)

Cg1 and Cg2 are the centroids of the C8–C13 and N1/C1–C5 rings, respectively.

**Table:**

*D*—H···*A*	*D*—H	H···*A*	*D*···*A*	*D*—H···*A*
C15—H15*B*···*Cg*1^ii^	0.96	2.82	3.679 (2)	149
C12—H12···*Cg*2^ii^	0.96	3.12	4.104 (2)	161

Symmetry code: (ii) −*x*, −*y*+1, −*z*+1.
